# ANO6 is a reliable prognostic biomarker and correlates to macrophage polarization in breast cancer

**DOI:** 10.1097/MD.0000000000036049

**Published:** 2023-11-10

**Authors:** Long-Huan Tang, Min Dai, Dong-Hai Wang

**Affiliations:** a General Surgical Department One, FengHua People’s Hospital, Ningbo, China; b Department of General Surgery, Hai’an Hospital Affiliated to Nantong University, Hai’an, China.

**Keywords:** ANO6 expression, biomarker, breast cancer, macrophage polarization

## Abstract

To investigate the value of Anoctamin 6 (ANO6) in breast cancer (BC) by analyzing its expression, prognostic impact, biological function, and its association with immune characteristics. We initially performed the expression and survival analyses, followed by adopting restricted cubic spline to analyze the nonlinear relationship between ANO6 and overall survival (OS). Stratified and interaction analyses were conducted to further evaluate its prognostic value in BC. Next, we performed enrichment analyses to explore the possible pathways regulated by ANO6. Finally, the correlations between ANO6 and immune characteristics were analyzed to reveal its role in immunotherapy. Lower ANO6 expression was observed in BC than that in the normal breast group, but its overexpression independently predicted poor OS among BC patients (*P* < .05). Restricted cubic spline analysis revealed a linear relationship between ANO6 and OS (P-Nonlinear > 0.05). Interestingly, menopause status was an interactive factor in the correlation between ANO6 and OS (P for interaction = 0.016). Additionally, ANO6 was involved in stroma-associated pathways, and its elevation was significantly linked to high stroma scores and macrophage polarization (*P* < .05). Moreover, ANO6 was notably correlated with immune checkpoint expression levels, and scores of tumor mutation burden and microsatellite instability (all *P* < .05). ANO6 was an independent prognostic factor for BC, and might be a potential target for the BC treatment. Besides, ANO6 might affect BC progression via the regulation of stroma-related pathways and macrophage polarization.

## 1. Introduction

Breast cancer (BC) is one of the most prevalent malignancies and is the second leading cause of death among females globally.^[1]^ It often occurs in postmenopausal women,^[2]^ which is categorized into luminal, human epidermal growth factor receptor 2+, and triple-negative BC (TNBC) according to estrogen receptor, progesterone receptor, and human epidermal growth factor receptor 2 status.^[3]^ Luminal subtype which expresses estrogen receptor accounts for approximately 70% of all BC cases.^[4]^ BC is a heterogeneous disease, which affects the same anatomical organ but with different etiology, clinical manifestations, and prognosis.^[5]^ Despite the fact that considerable improvements in screening and therapeutic methods have been made, tumor heterogeneity results in huge individual differences in the clinical outcome of BC treatment.^[6]^ Various studies have indicated that many biomarkers participated in the BC progression, but are the tip of the iceberg.^[7–9]^ Therefore, it is essential to develop more effective biomarkers for BC diagnosis and treatment.

Anoctamin 6 (ANO6), also known as TMEM16F, belongs to the anoctamin family containing 10 proteins (ANO1-10),^[10]^ and each protein has 8 transmembrane domains and cytosolic amino- and carboxyl-termini.^[11]^ As a plasma membrane, ANO6 is located at a Ca^2+^-activated anion with a relatively high cation permeability.^[12]^ Although ANO6 has multiple similar sequences in the transmembrane structure to ANO1, it has significantly different properties from those of ANO1, such as a notably higher EC_50_ for Ca^2+^.^[12,13]^ A previous study has revealed the involvement of ANO6 in blood coagulation and congenital bleeding disorder Scott syndrome.^[14]^ Besides, ANO6 has been reported to participate in cell migration, cell volume regulation, as well as externalization of phosphatidylserine.^[15]^ It has also been demonstrated that ANO6 promoted migration and invasion of glioma cells via regulation of the extracellular signal-regulated kinase signaling pathway.^[16]^ However, the role of ANO6 in BC is still elucidated.

This study aimed to investigate the important value of ANO6 as a prognostic biomarker for BC based on various bioinformatics databases. The mRNA and protein expression levels of ANO6 in BC and normal breast tissues were initially assessed. Then, the independent prognostic value of ANO6 was examined, followed by conducting restricted cubic spline (RCS) analysis to exhibit the nonlinear relationship between ANO6 and overall survival (OS). Finally, the enrichment analyses were conducted and the association of ANO6 with immune characteristics was assessed to investigate the potential mechanism of ANO6 in BC.

## 2. Materials and methods

### 2.1. Expression analysis of ANO6

The normalized RNA sequencing data based on TCGA Pan-Cancer were obtained from the UCSC Xena database (https://xenabrowser.net/datapages/) and the ANO6 gene expression levels were extracted to analyze the ANO6 expression levels in various cancers and normal samples. Tumor samples with less than 3 were deleted. Then, the differential gene expression of ANO6 in BC and normal groups was evaluated by the unpaired and paired t-tests. Besides, immunohistochemistry analysis was performed to assess ANO6 protein level in BC and normal breast tissues following the manufacturer’s protocol. Briefly, formalin-fixed tissue samples were embedded in paraffin and cut into 4 µm-thick sections which were then deparaffinized, rehydrated, and subjected to antigen retrieval and serum blocking. Next, the slides were incubated with ANO6 antibody (20784-1-AP, Proteintech) at 4°C overnight, followed by exposure to a secondary antibody for 1 hour at room temperature. After adding substrate and hematoxylin staining, slides were covered and observed under a microscope.

Following this, the transcriptome profiles (HTseq-FPKM), phenotype information, and survival data of TCGA-BC samples were downloaded using the UCSC Xena database. Inclusion criteria: BC samples; patients with matched survival and expression data. The adjacent normal breast samples were excluded (n = 114) and those with incomplete survival and expression information were excluded (n = 11). Finally, 1092 BC samples were enrolled for the study. All samples were divided into low and high ANO6 expression groups by the median value of ANO6. We then evaluated the associations of ANO6 expression with clinical characteristics.

### 2.2. Survival analysis of ANO6

The correlation between ANO6 and OS for BC patients was examined by using the Kaplan–Meier plotter method and the survival difference between the two groups was compared by the logrank test. Cox regression analysis was conducted to determine the prognostic role of ANO6 for OS among BC patients using 2 models and the hazard ratio and 95% confidence interval in the models were evaluated: with no covariates adjusted in a univariate model; with age, gender, histologic subtype, menopause status, race, and cancer stage adjusted in a multivariate model. RCS was adopted to assess the nonlinear relationship between ANO6 and OS. If there was nonlinearity, the inflection point was calculated using a recursive algorithm to construct a 2-piece linear regression model. Moreover, stratified and interaction analyses were conducted according to age, gender, histologic subtype, menopause status, race, and cancer stage.

### 2.3. Functional enrichment analysis

To investigate the biological function of ANO6 in BC, we performed Gene Ontology (GO) annotations and Kyoto Encyclopedia of Genes and Genomes (KEGG) pathway analyses using clusterProfiler package in R.^[17]^ GO annotations include cellular component, molecular function, and biological process. The co-expressed genes were first obtained in LinkedOmics (http://linkedomics.org/login.php). The selection filter was set as follows: cancer cohort: TCGA_BRCA; search dataset: TCGA_BRCA (RNAseq); search dataset attribute: ANO6; target dataset: TCGA_BRCA (RNAseq), and statistical method: Pearson Correlation test. The ANO6 association result will be presented in a volcano plot. The 50 positively and negatively significant genes were separately exhibited in a heat map. The top 500 identified co-expressed genes with *P*-value < 0.001 and FDR < 0.001 were selected for further GO and KEGG pathway analyses.

To further elucidate the underlying mechanism of ANO6 involved in BC, gene set enrichment analysis (GSEA) was performed to explore ANO6-related positive KEGG pathways using the following settings: number of permutations = 1000, permutations type = gene_set, enrichment statistic = weighted, metric for ranking genes = Pearson, genes list ordering mode = descending.^[18]^

### 2.4. Correlation between ANO6 and immune characteristics

The ESTIMATE algorithm is a method to understand the landscape of stromal and immune cells in the tumor microenvironment through gene expression level.^[19]^ This algorithm was used to calculate the stromal score, immune score, and Estimate score, and their associations with ANO6 were determined via Pearson analysis. CIBERSORT is a deconvolution algorithm based on RNA-seq data, which can estimate the infiltration levels of 22 immune cells in each sample.^[20]^ This algorithm was adopted to calculate the immune infiltration scores of 22 subtypes of immune cells in each sample. Besides, the patients were divided into low and high ANO6 groups by its median expression and we compared the differential distributions of 22 immune cell infiltration levels in 2 expression groups.

Since immune checkpoint inhibitors targeting CTLA4, PD-1, and PD-L1 represent a critical role in cancer treatment and immuno-therapy,^[21]^ we further evaluated the correlation between the expression levels of 3 immune checkpoints with ANO6 mRNA level by the Pearson correlation test.

Tumor mutation burden (TMB) and microsatellite instability (MSI) are useful predictors associated with a good response to immune checkpoint inhibitors.^[22]^ Pearson correlation test was also adopted to assess the association of ANO6 expression with TMB and MSI scores.

### 2.5. Statistical analysis

Statistical analyses were performed by SPSS version 23.0 and R version 4.2.2 software. Categorical variables were expressed as counts (percentages) and comparisons between 2 groups were analyzed using the Chi-square test or Fisher exact test. Continuous variables were represented as mean ± standard deviation or median [interquartile range]. The difference in continuous variables between the 2 groups was compared using the Student *t* test (normal distribution) or Mann-Whitney U-test (skewed distribution). The differences in continuous variables among at least 3 groups were compared using the one-way analysis of variance (normal distribution) or the Kruskal–Wallis rank sum test (skewed distribution). Univariate logistic regression analysis was performed to evaluate the correlation between ANO6 and clinicopathological characteristics in BC: luminal was set as a reference level for histologic subtype, premenopause for menopause status, African-American for race, and stage 1 for cancer stage. *P* < .05 was considered to be statistically significant.

## 3. Results

### 3.1. ANO6 expression in BC

**Figure 1.**
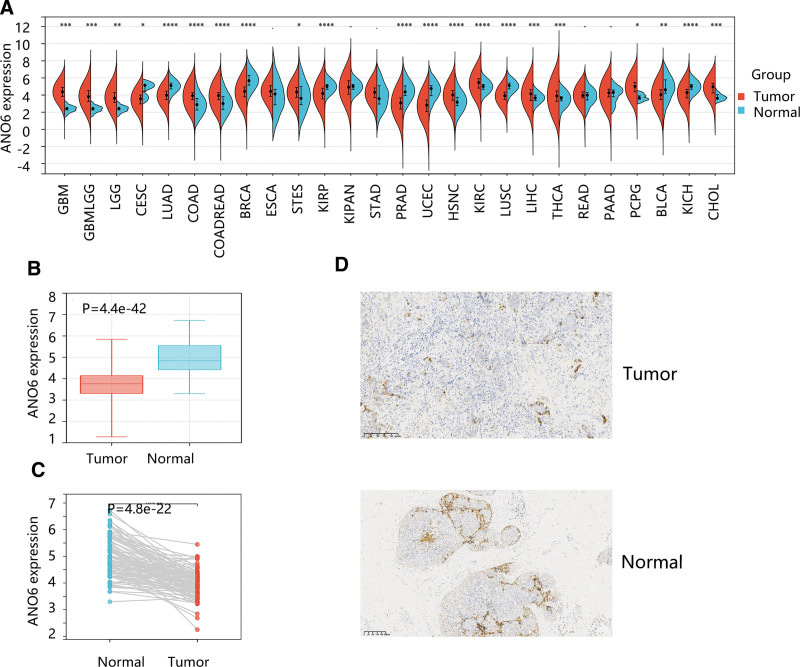
General profile of ANO6 expression in cancer. (A) ANO6 gene expression in various cancers. The differential gene expression of ANO6 in breast cancer and normal breast group using (B) unpaired and (C) paired *t*-tests. (D) The protein levels of ANO6 in breast cancer and normal breast tissues. *P < .05, **P < .01, ***P < .001. ANO6 = Anoctamin 6, TNBC = triple negative breast cancer.

First, we have an overview of the ANO6 gene expression in pan-cancer and found a significant difference in ANO6 expression in various cancers compared with the normal group (Fig. 1A), indicating that ANO6 might be related to tumor occurrence. Both unpaired and paired t-tests exhibited a significantly lower mRNA level of ANO6 in BC compared to the normal breast group (*P* < .05) (Fig. 1B and C). The ANO6 protein levels in BC and normal breast tissue are shown in Figure 1D.

**Figure 2.**
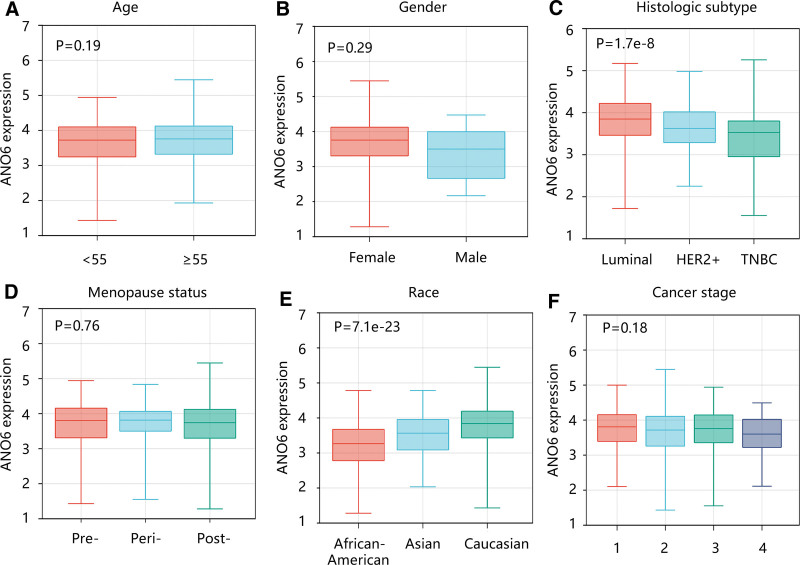
The association of ANO6 gene expression with clinical characteristics. (A) Age. (B) Gender. (C) Histologic subtype. (D) Menopause status. (E) Race. (F) Cancer stage. ANO6 = Anoctamin 6, TNBC = triple-negative breast cancer.

Following this, the relationship between ANO6 and clinical parameters was qualitatively analyzed. There were significant differences in the distribution of histologic subtype and race between the 2 ANO6 expressers (*P* < .001), but age, gender, menopause status, and cancer stage in the low ANO6 group were not significantly different from those in the high ANO6 group (*P* > .05) (Table 1). Similarly, the univariate logistic regression analysis results showed that TNBC, Asian, and Caucasian were significantly related to ANO6 expression (*P* < .05) (Table 2). When ANO6 was transformed into a continuous variable, the same results were observed in the quantitative analysis (Fig. 2A–F). In detail, there was no significant difference in ANO6 expression in different groups according to age, gender, menopause status, and cancer stage. Notably, patients with luminal BC tended to have the highest ANO6 mRNA expression compared with other subtypes (*P* < .01); and African–Americans had the lowest ANO6 mRNA expression than Caucasians and Asians (*P* < .01).

**Table 1:** Relationship between ANO6 levels and clinicopathological parameters of breast cancer patients.

Variables	ANO6		*χ* ^2^	*P* value
	Low (%)	High (%)		
Age (yr)			0.724	.395
<50	156 (28.6)	144 (26.3)		
≥50	389 (71.4)	403 (73.7)		
Gender			0.344	.578
Female	538 (98.7)	542 (99.1)		
Male	7 (1.3)	5 (0.9)		
Histologic subtype			34.150	<.001
Luminal	240 (44.0)	327 (59.8)		
HER2+	20 (3.7)	16 (2.9)		
TNBC	80 (14.7)	36 (6.6)		
Unknown	205 (37.6)	168 (30.7)		
Menopause status			3.321	.345
Premenopause	107 (19.6)	121 (22.1)		
Perimenopause	18 (3.3)	22 (4.0)		
Postmenopause	350 (64.2)	350 (64.0)		
Unknown	70 (12.8)	54 (9.9)		
Race			74.277	<.001
African–American	141 (25.9)	39 (7.1)		
Asian	35 (6.4)	26 (4.8)		
Caucasian	330 (60.6)	427 (78.1)		
Unknown	39 (7.2)	55 (10.1)		
Cancer stage			3.588	.465
Stage 1	82 (15.0)	97 (17.7)		
Stage 2	323 (59.3)	300 (54.8)		
Stage 3	120 (22.0)	127 (23.2)		
Stage 4	11 (2.0)	9 (1.6)		
Unknown	9 (1.7)	14 (2.6)		

ANO6 = Anoctamin 6, HER2+ = human epidermal growth factor receptor 2+, TNBC = triple-negative breast cancer.

**Table 2:** Correlation between the ANO6 expression and clinical parameters using univariate logistic regression analysis.

Variables	Odds ratio	95% confidence interval	*P* value
Age	1.007	0.998–1.016	.150
Gender	0.709	0.224–2.248	.559
HER2+	0.587	0.298–1.157	.124
TNBC	0.330	0.215–0.506	<.001
Perimenopause	1.081	0.550–2.123	.821
Postmenopause	0.884	0.656–1.193	.421
Asian	2.686	1.446–4.989	.002
Caucasian	4.678	3.191–6.858	<.001
Stage 2	0.785	0.563–1.096	.155
Stage 3	0.895	0.608–1.316	.572
Stage 4	0.692	0.273–1.751	.437

ANO6 = Anoctamin 6, HER2+ = human epidermal growth factor receptor 2+, TNBC = triple-negative breast cancer.

### 3.2. Prognostic value of ANO6 for BC

**Figure 3.**
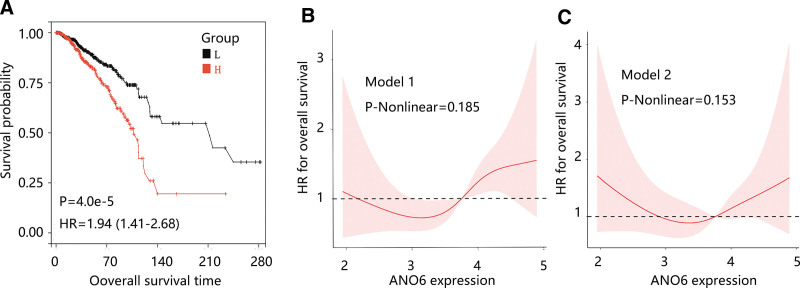
The association of ANO6 with OS in patients with breast cancer. (A) High ANO6 expression led to shorter OS. Restricted cubic spline revealed the linear relation between ANO6 and OS in model 1 (B) and model 2 (C). Model 1: no covariates were adjusted; model 2: covariates including age, gender, histologic subtype, menopause status, race, and cancer stage were adjusted. ANO6 = Anoctamin 6, OS = overall survival.

To investigate the prognostic role of ANO6 in BC, we explored the association of ANO6 with OS. Patients with high ANO6 expression had shorter survival with statistical significance (*P* < .05) (Fig. 3A). Further Cox regression analysis showed that high ANO6 expression was remarkably linked to unfavorable clinical outcomes in the univariate and multivariate models (*P* < .05) (Table 3). When the ANO6 was coded into 2 groups, ANO6 was still significantly linked to higher death risk among BC patients (*P* < .05) (Table 3). RCS analysis revealed a linear association of ANO6 with OS in the unadjusted model 1 (P-Nonlinear = 0.185) and model 2 (P-Nonlinear = 0.153) (Fig. 3B and C). These results indicated that ANO6 upregulation was an independent predictor for poor OS in patients with BC and ANO6 had a linear connection to OS.

**Table 3:** Cox regression analysis of ANO6 and other clinical parameters for breast cancer.

	Univariate model		Multivariate model	
	HR (95% CI)	*P* value	HR (95% CI)	*P* value
ANO6 expression	1.957 (1.459–2.624)	<.001	2.064 (1.285–3.317)	.003
ANO6 group (high vs low)	1.849 (1.330–2.572)	<.001	2.525 (1.413–4.510)	.002

Univariate model: no covariates adjusted; Multivariate model: all covariates including age, gender, histologic subtype, menopause status, race, and cancer stage were adjusted.

95% CI = 95% confidence interval, HR = hazard ratio.

**Figure 4.**
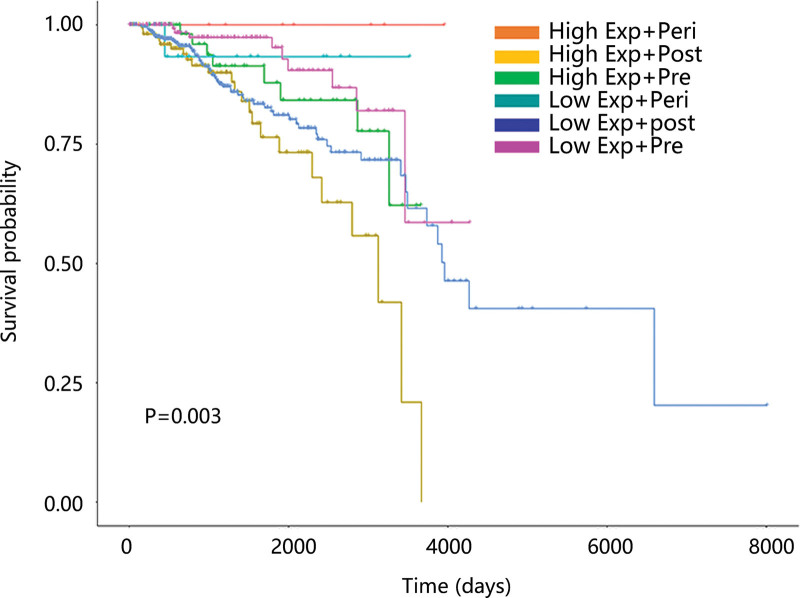
The correlation between ANO6 combined with menopause status and overall survival of patients with breast cancer. ANO6 = Anoctamin 6.

In the subgroup analysis, high ANO6 expression was significantly related to worse OS in age ≥ 50 years, female, luminal subtype, postmenopause, Caucasian, and stage 1 + 2 subjects (all *P* < .05). No significant interaction was observed except for menopause status (P for interaction = 0.016) (Table 4). Therefore, we explored the relationship between ANO6 combined with menopause status and the OS of BC patients. Expectedly, different ANO6 expression patterns combined with menopause status had a close relation with OS (*P* < .05) (Fig. 4). Compared with high ANO6 mRNA expression + postmenopause patients, high ANO6 mRNA expression + perimenopause patients had a higher survival probability.

**Table 4:** The association of ANO6 with overall survival among different subgroups.

Subgroups	*P* value	*P* for interaction	Hazard ratio	Lower	Upper
Age		0.259			
<50	.120		1.63	0.87	3.05
≥50	<.001		2.14	1.47	3.14
Gender		0.484			
Female	<.001		1.96	1.42	2.71
Male	/		/	/	/
Histologic subtype		0.985			
Luminal	.005		2.17	1.24	3.78
HER2+	.400		0.41	0.05	3.54
TNBC	.190		1.86	0.72	4.83
Menopause status		0.016			
Premenopause	.390		1.50	0.59	3.82
Perimenopause	/		/	/	/
Postmenopause	.001		1.96	1.29	2.97
Race		0.885			
African–American	.160		0.54	0.22	1.30
Asian	/		/	/	/
Caucasian	<.001		2.35	1.59	3.49
Cancer stage		0.179			
Stage 1 + 2	<.001		2.32	1.49	3.61
Stage 3 + 4	.220		1.38	0.82	2.31

ANO6 = Anoctamin 6, HER2+ = human epidermal growth factor receptor 2+, TNBC = triple-negative breast cancer.

### 3.3. Biological function of ANO6

**Figure 5.**
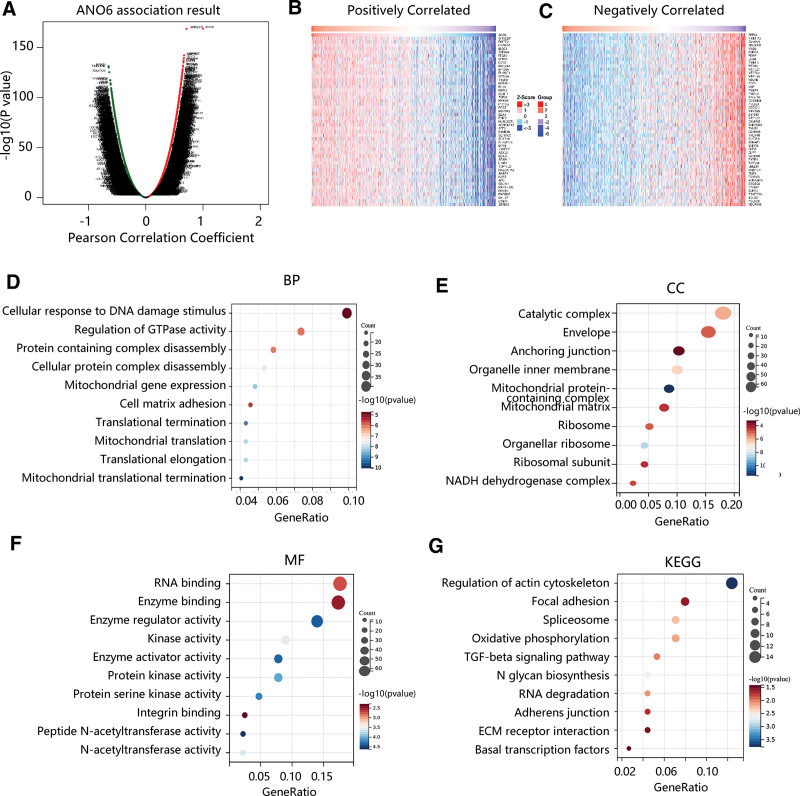
Co-expressed genes analysis associated with ANO6 in breast cancer. (A) The volcano plot of ANO6-associated genes. (B) Fifty positively correlated significantly genes with ANO6. (C) 50 negatively correlated significantly genes with ANO6. (D) Biological process. (E) Cellular component. (F) MF. (G) KEGG pathway analysis. KEGG = Kyoto Encyclopedia of Genes and Genomes, MF = molecular function.

**Figure 6.**
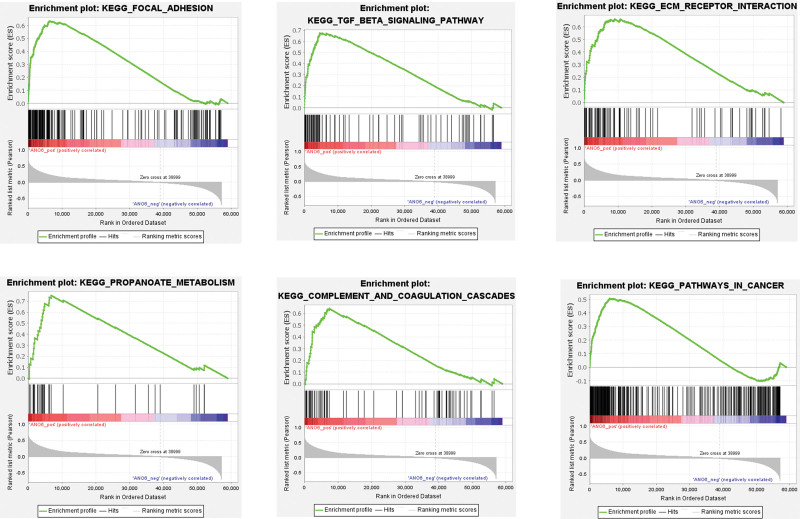
Gene set enrichment analysis revealed the top 6 pathways positively associated with ANO6. ANO6 = Anoctamin 6.

After determining the potent value of ANO6 in BC, its possible mechanism was subsequently explored. The ANO6 co-expressed genes in BC and the association result was presented in the volcano plot, displaying that there were 7323 genes (green dots) significantly negatively correlated with ANO6 and 8155 genes (red dots) had significant positive correlations with ANO6 (Fig. 5A). Heat maps exhibited the top 50 significant genes positively and negatively correlated with ANO6, respectively (Fig. 5B and C). As for biological process, the genes were mainly enriched in cellular response to DNA damage stimulus, regulation of GTPase activity, and cell matrix adhesion (Fig. 5D). For cellular component, the genes were mainly involved in catalytic complex, anchoring junction, and organelle inner membrane (Fig. 5E). Molecular functions were RNA binding, enzyme binding, and enzyme regulator activity (Fig. 5F). The major KEGG pathways that ANO6 participated in included regulation of actin cytoskeleton, focal adhesion, spliceosome, adherens junction, and ECM receptor interaction (Fig. 5G). Moreover, GSEA showed that ANO6 was positively associated with focal adhesion, TGF-beta signaling pathway, ECM receptor interaction, propanoate metabolism, complement and coagulation cascades, and pathways in cancer (Fig. 6). The combined KEGG with GSEA results revealed that ANO6 might affect BC progression through the activation of these stroma-associated pathways.

### 3.4. Role of ANO6 in immune therapy

**Figure 7.**
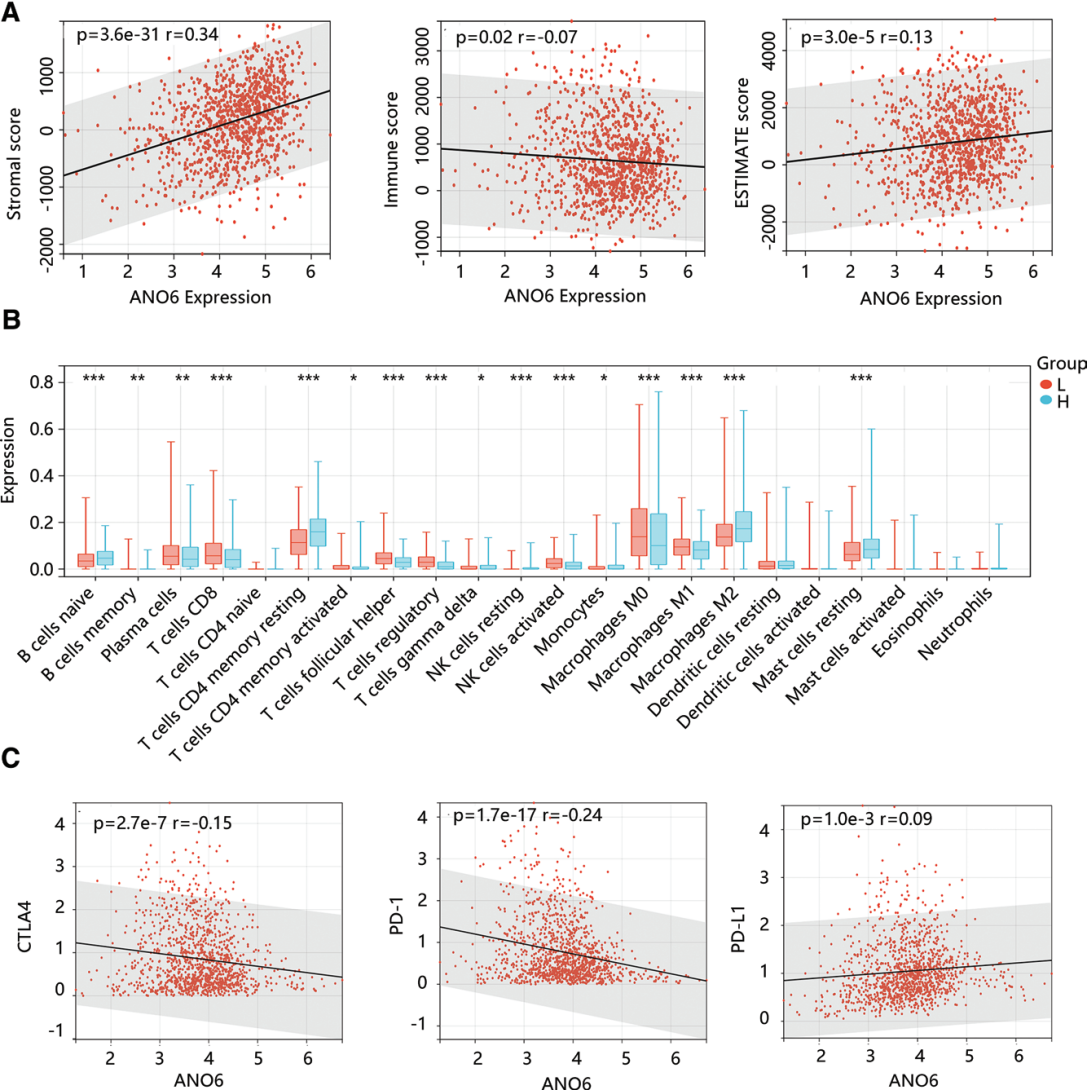
Immune cell infiltration analysis in breast cancer. (A) The association of ANO6 with stromal and immune scores using the ESTIMATE algorithm. (B) The distribution of various immune cells in different ANO6 groups using the CIBERSORT algorithm. (C) The correlation between ANO6 and immune checkpoints’ expression levels. ANO6 = Anoctamin 6.

**Figure 8.**
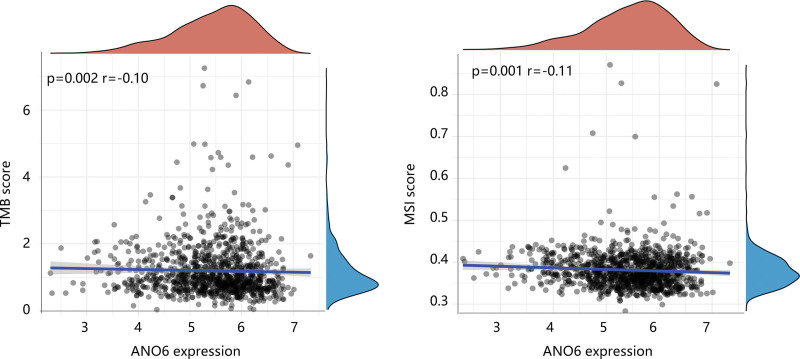
The association of ANO6 with tumor mutation burden score and microsatellite instability score. ANO6 = Anoctamin 6, MSI = microsatellite instability score, TMB = tumor mutation burden.

Since the essential role of cancer immunology and immune therapy, we explored the possible impact of ANO6 on different immune cell types in the BC microenvironment. As shown in Figure 7A, ANO6 had a strong positive connection to stromal score and Estimate score, and a significant negative correlation between ANO6 and immune score was observed (all *P* < .05). In addition, the abundance of various immune cells including B cells memory, plasma cells, T cells CD8, T cells CD4 memory activated, T cells follicular helper, T cells regulatory, T cells gamma delta, NK cells activated, monocytes, macrophages M0, macrophages M1, and macrophages M2 in the low ANO6 group was significantly different from those in the high ANO6 group (Fig. 7B). Notably, the macrophage M1 infiltration level was low, while the macrophage M2 infiltration level was high in the high-ANO6 group (*P* < .05). This suggested that ANO6 overexpression might promote the polarization of macrophages, which has an intimate correlation with the immunosuppressive state of the tumor.^[23]^ Besides, ANO6 had an obvious negative association with CTLA4 (*r* = −0.15, *P* < .05) and PD-1 (*r* = −0.24, *P* < .05), but was slightly positively correlated to PD-L1 (*R* = 0.09, *P* < .05) (Fig. 7C). Moreover, patients with high ANO6 expression tended to have low scores of TMB and MSI (all *P* < .05) (Fig. 8). These findings indicated that BC patients with high ANO6 expression might be less sensitive to immunotherapy.

## 4. Discussion

Herein, our results revealed an intimate association of ANO6 with BC. We initially observed downregulated expression levels of ANO6 in BC and ANO6 overexpression serves as an independent predictor for worse prognosis of BC patients. Therefore, ANO6 downregulation was associated with BC occurrence, but high ANO6 expression might contribute to BC progression. Notably, patients with high ANO6 mRNA expression + perimenopause had a higher survival probability than those with high ANO6 mRNA expression + postmenopause. Low expression of CYP34A1 had a poor prognosis in BC, while patients at postmenopause had the lowest CYP34A1 expression compared with other groups, indicating that menopause status might have an impact on the prognosis of BC patients,^[24]^ which is similar to our results. The clinicians could benefit from the prognostic biomarker ANO6 expression to identify high-risk groups of BC, guide the optimal treatment intervention mode, optimize patient management, and improve patient prognosis.

We next focused on the possible pathways regulated by ANO6 in BC. Enrichment analysis revealed focal adhesion, TGF-beta signaling pathway, and ECM receptor interaction were the consistent pathways. Cell adhesion molecules can mediate cell adhesion, control cell motility, and conduct intracellular signals.^[25]^ Besides, focal adhesion can control cell morphology, adhesion, and migration by linking ECM and intracellular F-actin.^[26]^ These are essential for cancer invasion, metastasis, and resistance to cancer therapy.^[27]^ Ning et al demonstrated the involvement of the focal adhesion signaling pathway in the process of epithelial-to-mesenchymal transition (EMT) in pancreatic cancer.^[28]^ Focal adhesion pathway can alter cell glycolysis and induce cisplatin resistance in breast cancer.^[29]^ In early-stage tumors, the TGF-beta pathway is a tumor suppressor to induce cancer cell apoptosis and inhibit cancer cell proliferation. Instead, in late-stage, it exerts pro-tumor effects by regulating genomic instability, EMT, neovascularization, cell motility, immune invasion, and metastasis.^[30]^ ECM components provide cells with biochemical and biomechanical cues and represent a central role in BC cancer progression and metastasis.^[31,32]^ Therefore, the authors speculated that ANO6 might affect BC progression and unfavorable clinical outcomes by regulating these key pathways.

Subsequently, immune cell infiltration analysis was conducted to further determine the underlying mechanism of ANO6 in BC. We observed a strong positive correlation between ANO6 and stromal score and found a differential abundance of various immune cells in 2 different ANO6 expression groups. The surrounding stromal cells, including cancer-associated fibroblasts (CAFs), can act as secretors of active cytokines and chemokines, affecting the infiltration of tumor-infiltrating lymphocytes and the balance of local inflammatory response.^[33,34]^ CAFs coordinate the promotion of tumor inflammation and regulate TIME toward immunosuppression. These functions are mediated through intricate reciprocal signaling interactions with cancer cells, stromal components, and infiltrating immune cells. Additionally, CAFs may recruit regulatory T cells and myeloid cells, promoting polarization and infiltration of M2 macrophages.^[33]^ Myeloid cells have the ability to secrete anti-inflammatory cytokines such as TGF-beta and IL-10 and drive macrophages toward the M2 phenotype.^[35]^ Epidermal growth factor (EGF) expressed by macrophages M2 activated the downstream molecules to avoid the cancer cell apoptosis but promote its proliferation.^[36]^ Besides, macrophages M2 was involved in the epithelial-mesenchymal transition process by reducing E-cadherin expression and participating in the TGF-beta/Smad2 pathway and IL-10 pathway, thereby promoting cancer cell metastasis.^[37]^ In addition, macrophages M2 can produce growth factors and pro-angiogenic cytokines to initiate the metastasis process.^[38]^ Compared with macrophages M1, macrophages M2 are less sensitive to antiangiogenic and radiation therapies.^[39,40]^ Interestingly, our findings revealed that ANO6 overexpression promotes the polarization of macrophages to M2. Thus, it is reasonable to infer that the upregulation of ANO6 was closely related to high levels of stromal cells, accelerating macrophages to polarize to an M2-like phenotype, and hence leading to BC growth and development.

Finally, we found that ANO6 was significantly correlated with immune checkpoint expressions and there were negative associations of ANO6 with TMB and MSI scores. Cancer cells escape immune surveillance by altering their surface antigens to evade detection and destruction by lymphocytes.^[41]^ Besides, they can escape immune recognition by modulating the immune checkpoint expression.^[42]^ The abnormal expression of immune checkpoint expression inhibits the ability of cytotoxic T-cells to eliminate cancer cells.^[43]^ Therefore, ANO6 elevation might affect tumor immune microenvironment and accelerate tumor immune escape. High TMB were more enriched with favorable immune infiltrates, including the presence of activated cells, CD4 + T cells, and CD8 + T cells,^[44]^ which was consistent with our findings that the infiltration levels of these 3 cells were significantly lower in the high ANO6 group. Thus, patients with high ANO6 expression and low TMB, resulting in less sensitive to immunotherapy and shorter survival.

For strengths, this is the first study comprehensively investigating the value of ANO6 in BC including its expression patterns, survival analysis, enrichment analysis, and immune cell infiltration analysis in a large-scale cohort. RCS analysis revealed a linear relationship between ANO6 and OS. Moreover, interaction analysis exhibited that menopause status was an interactive factor in the correlation between ANO6 and OS. However, the complex interaction mechanism requires further investigation. Although this study provides a potential robust biomarker for guiding the BC treatment and improving the prognosis of BC patients, our results should be verified by in vitro and in vivo experiments as well as in our own cohort in future research.

In conclusion, elevated ANO6 level is an independent factor for predicting poor OS in BC patients. Moreover, ANO6 might affect BC progression by activating stroma-related pathways and promoting macrophage polarization, which should be validated by experiments. This study revealed that ANO6 might be a potential biomarker to improve the prognosis of BC patients.

## Author contributions

**Conceptualization:** Long-Huan Tang.

**Data curation:** Long-Huan Tang, Min Dai.

**Investigation:** Min Dai, Dong-Hai Wang.

**Visualization:** Long-Huan Tang, Dong-hai Wang.

**Writing – original draft:** Long-Huan Tang, Min Dai.

**Writing – review & editing:** Long-Huan Tang.
